# Supplementary material for: The Moderating Role of the School Context on the Effects of the Healthy Primary School of the Future
Source: Int J Environ Res Public Health. 2019 Jul 9;16(13):2432. doi: 10.3390/ijerph16132432 (PMC6651395; doi:10.3390/ijerph16132432)
Supplement: Supplementary file 1 [file ijerph-16-02432-s001.pdf]

Supplementary Material

Table 1. School-specific intervention effects of HPSF after two years' follow-up.

	School 1 vs Control			School 2 vs Control			School 3 vs Control			School 4 vs Control		
	**B (95% C.I.)**	***p***	**ES**	**B (95% C.I.)**	***p***	**ES**	**B (95% C.I.)**	***p***	**ES**	**B (95% C.I.)**	***p***	**ES**
**BMI z-score**	−0.107 (−0.18 − −0.03)	**0.01**	−0.11	−0.052 (−0.14 − 0.04)	0.24	−0.05	−0.045 (−0.13 − 0.04)	0.30	−0.04	−0.080 (−0.16 − 0.00)	**0.04**	−0.08
**Sedentary** (% per day)	−1.493 (−2.77 − −0.21)	**0.02**	−0.27	−0.461 (−2.03 − 1.10)	0.56	−0.08	−0.141 (−1.71 − 1.43)	0.86	−0.03	−0.437 (−1.74 − 0.86)	0.51	−0.08
**Light** (% per day)	1.145 (0.13 − 2.16)	**0.03**	0.26	0.370 (−0.88 − 1.62)	0.56	0.09	0.061 (−1.18 − 1.31)	0.92	0.01	0.066 (−0.97 − 1.10)	0.90	0.02
**MVPA** (% per day)	0.355 (−0.18 − 0.89)	0.19	0.15	0.120 (−0.53 − 0.77)	0.72	0.05	0.098 (−0.56 − 0.75)	0.77	0.04	0.347 (−0.20 − 0.89)	0.21	0.15
**Healthy dietary behaviours** (mean days/week)	0.251 (0.06 − 0.45)	**0.01**	0.25	0.032 (−0.24 − 0.30)	0.82	0.03	0.083 (−0.16 − 0.33)	0.50	0.08	−0.080 (−0.28 − 0.12)	0.44	−0.08
**Unhealthy dietary behaviours** (mean days/week)	−0.085 (−0.24 − 0.07)	0.28	−0.13	−0.044 (−0.26 − 0.17)	0.69	−0.07	0.039 (−0.15 − 0.23)	0.70	0.06	−0.013 (−0.17 − 0.15)	0.88	−0.02
**School water consumption** (0-3)	1.161 (0.91 − 1.42)	**<0.01**	1.16	1.176 (0.90 − 1.46)	**<0.01**	1.17	−0.197 (−0.49 − 0.10)	0.19	−0.20	0.137 (−0.11 − 0.39)	0.28	0.14
**Minimum of two food types during lunch** (% yes)	2.52 (1.22 − 5.18) *	**0.01**	na	3.96 (1.43 − 10.94) *	**0.01**	na	0.20 (0.08 − 0.53) *	**<0.01**	na	0.94 (0.52 − 1.69) *	0.84	na

* Presented as odds ratio. Abbreviations: CI = confidence interval; *p* = *p*-value; ES = effect size; PA = physical activity; MVPA = moderate to vigorous physical activity; na = not applicable.

Table 2. Explanation of the nutrition-related practices of teachers.

**Healthy modelling**	I consciously eat healthy food products when the children are around.<br>*For example, by eating fruit when the children are around.*
**Prevent unhealthy modelling**	I consciously do not eat unhealthy food products when the children are around.<br>*For example, by eating unhealthy food products in the teachers' room or at home.*
**Encouragement**	I encourage the children to eat healthily.<br>*For example, by encouraging the children to bring and eat vegetables as a midmorning break. Or by encouraging them to first eat their lunch and afterwards their sweet snack.*
**Involving**	I involve the children in things that concern a healthy diet.<br>*For example, by involving the children when handing out fruit. By involving children in a vegetable garden. By letting children taste different sorts of fruit during the lesson.*
**Discussing**	I discuss nutrition with the children.<br>*For example, by discussing the food products they eat and like.*
**Educating**	I teach the children about nutrition.<br>*For example, by teaching the children where food comes from. By explaining that eating healthy foods is better than candy or cookies.*
**Providing feedback**	I give feedback to the children concerning their dietary behaviour.<br>*For example, by commenting when a child often does not finish his/her lunch, often eats unhealthy foods or does not take the time to eat.*
**Visibility**	I ensure that healthy food products are visible for the children (e.g. pictures).<br>*For example, by hanging up pictures of fruit in the classroom. Or by hanging up visualizations (e.g. mind map) of group sessions about healthy foods.*
**Routines**	I ensure that there are healthy habits during moments of eating and drinking in school.<br>*For example, by taking enough time for lunch and to wait till everyone is finished.*
**Monitoring**	I try to watch what the children are eating during the day.<br>*For example, by paying extra attention when a child often eats unhealthy foods as midmorning break or lunch.*
**Rules**	I strictly follow school policy on nutrition in school and in class.<br>*For example, by following strictly the policy for celebration treats or the prohibition on energy drinks.*
**Pressure to eat**	I insist that children finish their meal.<br>*For example, by insisting that children completely finish their lunch.*
**Instrumental feeding**	I reward the children sometimes with unhealthy food products when they did something well.<br>*For example, by rewarding the children with candy in a certain lesson when they did something really well.*

Table S2b. Explanation of the PA-related practices of teachers.

**Encouragement**	I encourage the children to be physically active.<br>*For example, by discussing the advantages of physical activity. By providing ideas for PA games during breaks and playing these games during PE.*
**Rewarding**	I reward the children for being physically active.<br>*For example, by giving the children compliments when they are very physically active during breaks or performed well during PE.*
**Involving**	I involve the children in things that concern physical activity.<br>*For example, by giving the children a choice in the kind of energizer for that moment, or the kind of activity during part of the PE.*
**Healthy modelling**	I am consciously physically active when the children are around.<br>*For example, by playing outside with the children during breaks, dancing together with the children, or do an energizer together. By commuting to school also in an active way.*
**Discussing**	I discuss physical activity with the children.<br>*For example, by discussing the different possibilities for physical activity that the children like or dislike. By asking which sports they play in their leisure time.*
**Educating**	I teach the children about physical activity.<br>*For example, by teaching the children new options to be physically active or teaching them the rules of games. By teaching the children that physical activity is good for your health.*
**Discouragement**	I correct children when they do not sit quietly during lessons.<br>*For example, when a child is moving on his chair continuously.*
**Providing feedback**	I give feedback to the children about their physical activity behaviour.<br>*For example, by discussing their physical activity behaviour and giving ideas for more ways of being physically active.*
**Availability**	I ensure there is PA-friendly equipment available for the children.<br>*For example, by preparing outdoor toys like a skipping rope or football.*
**Accessibility**	I enable the children to be physically active.<br>*For example, by regularly using energizers in the classroom. By always giving children the opportunity to be physically active even when it is raining outside.*
**Routines**	I ensure healthy PA habits in school.<br>*For example, by planning the times for energizers. By ensuring that the PA activity is fun and that everybody can join in.*
**Warning PA**	I warn the children about the possible risks of physical activity and playing outside.<br>*For example, by warning the children they can hurt themselves or get dirty when playing outside.*
**Monitoring**	I check in general the amount of physical activity of the children during a day.<br>*For example, by paying extra attention to someone who is not often physically active during breaks or PE.*
**Rules**	I strictly follow the school policy on physical activity in the school and in the classroom.<br>*For example, by strictly following the rules in the classroom or school yard regarding physical activity.*
**Pressure to be physically active**	I insist that the children be physically active.<br>*For example, by insisting that children always go outside during breaks, or that children always participate during PE or energizers.*
**Instrumental feeding**	I reward the children sometimes by watching a movie together or letting them be on the computer when they did well.<br>*For example, when it is almost holiday and they worked well in the last weeks.*
